# Substance Use, Self-Prescription and Burnout in Belgian Medical Doctors

**DOI:** 10.1192/j.eurpsy.2022.91

**Published:** 2022-09-01

**Authors:** G. Dom

**Affiliations:** University of Antwerp, Psychiatry, Boechout, Belgium

**Keywords:** alcohol, medical doctors, substance abuse, burnout

## Abstract

Alcohol, psychoactive substance abuse, and burnout are areas of significant problems within health care professionals and medical doctors. In 2011 we did a survey (n = 1601) in Belgium among the medical specialists. Beginning of 2021, exactly 10 years after the first survey, we repeated the survey this time among both medical specialists, general practitioners, and pharmacists. The results of this survey (N = 4361) will be presented and tendencies compared with international findings. We discuss the findings from both the longitudinal perspective using our 2011 data and also from the perspective of the impact of the Covid19 pandemic on health professionals.

**Disclosure:**

No significant relationships.

